# Catheter-Directed Thrombolysis in the Management of Thrombotic Peripheral Artery Occlusions—Acute and Mid-Term Clinical Outcomes

**DOI:** 10.3390/jcm13195732

**Published:** 2024-09-26

**Authors:** Ulrich Beschorner, Tanja Boehme, Elias Noory, Roaa Bollenbacher, Jonas Salm, Kambis Mashayekhi, Dirk Westermann, Thomas Zeller

**Affiliations:** 1 University Heart Center Freiburg-Bad Krozingen, Faculty of Medicine, University of Freiburg, Südring 15, 79189 Bad Krozingen, Germanyelias.noory@uniklinik-freiburg.de (E.N.); roaa.bollenbacher@uniklinik-freiburg.de (R.B.); jonas.salm@uniklinik-freiburg.de (J.S.); dirk.westermann@uniklinik-freiburg.de (D.W.); thomas.zeller@uniklinik-freiburg.de (T.Z.); 2 MediClin Herzzentrum Lahr, Hohbergweg 2, 77933 Lahr, Germany; kambis.mashayekhi@mediclin.de

**Keywords:** catheter-directed thrombolysis, peripheral artery disease, serious adverse events, bleeding, complication, amputation

## Abstract

**Objectives**: To evaluate the safety and efficacy of catheter-directed thrombolysis (CDT) with the recombinant tissue plasminogen activator (rt-PA) in all patients with symptomatic peripheral artery disease in real world practice. **Methods:** Consecutive patients treated with CDT between January 2013 and December 2020 were included in this retrospective analysis. The primary endpoint was the rate of serious adverse events (SAEs) until discharge. Secondary endpoints included interventional success, predictors for SAEs, bleeding and reperfusion edema/compartment syndrome, limb salvage, and clinical outcomes including target lesion revascularization rate (TLR). **Results:** Overall, 1238 patients were treated with CDT. SAEs occurred in 511 (41.3%) of the patients, 314 (25.4%) being bleeding complications. There were 95 cases of reperfusion edema/compartment syndrome. Forty-two patients underwent amputation and 33 patients (2.7%) died. CDT was successful in 1177 cases (95.1%). Multivariate logistic regression analysis identified age, abciximab and alprostadil usage, and lysis duration as predictors for SAEs and the use of abciximab as a predictor of reperfusion edema/compartment syndrome. Predictors for bleeding were age, alprostadil usage, and lysis duration. At 12 and 24 months, the limb salvage rate was 91.6% and 88.8%, and TLR rate was 46% and 57.2%, respectively. **Conclusions:** CDT is an effective endovascular method for the treatment of thrombotic peripheral artery occlusions but is associated with a high complication rate. For SAEs in general and bleeding specifically, increasing age, alprostadil use, and lysis duration were independent risk factors.

## 1. Introduction

In addition to aspiration and mechanical thrombectomy, catheter-directed thrombolysis (CDT) is an important treatment option for patients with embolic or local thrombotic arterial occlusions [1].

Besides urokinase, which is no longer available in many countries (recombinant) tissue plasminogen activator (rt-PA) is the most effective agent [2].

Randomized controlled studies comparing surgical therapy and CDT showed no significant difference in amputation-free survival and mortality at 6 and 12 months, respectively. However, more bleeding complications occurred in the CDT group [3,4].

After propensity matching, 10,484 patients were included in an analysis by Kolte et al. Patients with ALI were revascularized either surgically or underwent endovascular recanalization. Endovascular revascularization had significantly lower in-hospital mortality, composite of death/myocardial infarction/stroke, even major bleeding (16.7% vs. 21.0%; *p* < 0.001), and transfusion (10.3% vs. 18.5%; *p* < 0.001), but higher vascular complications (1.4% vs. 0.7%; *p* = 0.002), compared with those undergoing surgical revascularization [5]. A systemic review also showed CDT to be an effective therapy for the treatment of arterial occlusions. However, even in this review, bleeding complications were considered a substantial limitation of this therapy [6].

This retrospective study evaluates the safety and efficacy of the thrombolytic therapy with rt-PA in real world practice. The frequency, seriousness, and predictors of complications should be identified.

## 2. Methods

### 2.1. Patient Population

A consecutively collected and retrospectively evaluated registry was established to identify patients treated with CDT because of symptomatic peripheral vascular disease in various arterial locations including bypass grafts. Patients undergoing lysis therapy for upper extremity vascular occlusion were excluded. There were no other exclusion criteria. Patients treated between January 2013 and December 2020 were included in this analysis. A local ethics committee has provided study approval. The study is registered in the German Clinical Trials Register: DRKS00030930.

### 2.2. Study Endpoints

The primary endpoint was the rate of serious adverse events (SAEs) until discharge, defined as any complication requiring therapy, including surgery or other correction or prolonged hospital stay that occurred until discharge. SAEs are defined and classified according to ISO 14155:2020 [7] definitions (Supplement Table S1). In patients with multiple SAEs, only the most significant was documented. The complications were categorized as bleeding, reperfusion syndrome/compartment syndrome, amputation, death, and others. Major bleeding [8] was defined as a drop of hemoglobin of 4 g/dL if bleeding was not visible or of 3 g/dL if bleeding was evident. In addition, the administration of a blood transfusion in patients with a baseline hemoglobin level above 8 g/dL was defined as major bleeding and, therefore, an SAE.

The frequency of complications was investigated for the total cohort and for patient-subcohorts presenting with acute and chronic symptoms. Acute symptoms were defined as symptom onset within a maximum of 14 days prior to treatment. Symptoms were considered chronic if they had been present for more than 14 days.

Secondary endpoints included acute interventional success, i.e., the establishment of blood flow in the treated vessel area following CDT and potential adjunctive therapies defined as the complete removal of the occlusion or sufficient removal to reveal an underlying etiological factor that was responsible for the occlusion.

Further endpoints were predictors of complications: acute limb salvage as well as after 6, 12, and 24 months, survival rate, target lesion revascularization rate (TLR), change in clinical symptoms according to the Rutherford–Becker classification (RBC), and change in ankle–brachial index (ABI) after 6, 12, and 24 months.

### 2.3. Endovascular Technique

The decision to establishing CDT was left to the discretion of the operator and was provided via a multi-side-hole catheter (Cragg-McNamara™ Micro Therapeutics Infusion Catheter, Dublin, Ireland). In all cases, the thrombolytic agent used was rt-PA, and bolus dose and flow rate was determined individually. All patients received heparin through the sheath of the infusion catheter, controlled by activated partial thromboplastin time. In the case of heparin-induced thrombocytopenia in the medical history, agatroban infusions were administered. It was up to the physicians’ discretion whether or not abciximab or alprostadil had to be administered in addition.

The operator determined the duration of CDT. During thrombolysis, patients were monitored in the intensive care unit. Following CDT, a control angiography was performed the day after the index procedure or, if indicated, the same day and, depending on the outcome, further endovascular procedures were performed (e.g., balloon angioplasty or stent implantation) to finish the revascularization procedure successfully (<30% residual stenosis).

### 2.4. Statistical Analysis

For the analyses, SPSS software (version 25.0; SPSS, Chicago, IL, USA) was used. Continuous data were presented as means ± standard deviation, and categorical data were given as counts (percentages). Categorical variables were compared with the Fisher’s exact test, and continuous data were compared with the Student’s t-test.

For identification of the risk factor for serious adverse events, peri-procedural major bleeding and reperfusion edema/compartment syndrome, binary logistic regression analysis was used by means of a stepwise forward variable selection procedure. The following patient characteristics and treatment details were included in the analysis: age, body mass index, comorbidities, smoking status, further vascular diseases, intervention details, medication, lysis characteristics, and symptom duration (acute versus chronic). Results of the regression analysis are given as an odds ratio with 95% confident intervals, and *p*-values were 2-sided with a significance threshold of <0.05.

## 3. Results

### 3.1. Patient, Lesion, and Procedure Characteristics

**Figure 1:**
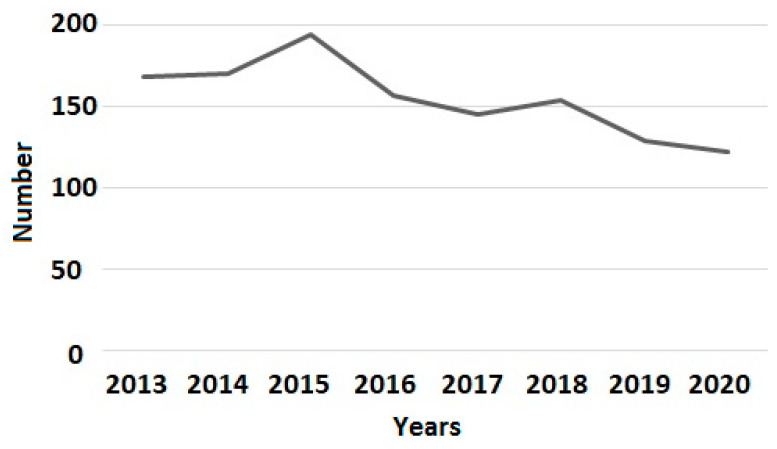
Incidence of CDT from 2013 to 2020. CDT—catheter-directed thrombolysis.

There was an overall decrease in use of CDT during the study period as shown in Figure 1.

Overall, 1238 patients were included in this study, consisting of 333 women (26.9%) and 905 men (73.1%) aged between 16 and 99 years. Baseline characteristics are shown in Table 1.

In 97.9% of patients, the baseline RBC was 3 or worse. A total of 589 patients (47.6%) suffered from pain at rest (RBC 4) and 233 patients (18.9%) suffered from ulcerations or necrosis (RBC 5 and 6). A total of 639 patients (51.6%) had acute symptoms, whereas 599 had chronic symptoms (48.4%). The grading of the symptoms is shown in Table 1. The mean age was 69.6 ± 12.2 years in the cohort with acute symptoms and 68.4 ± 11.4 years in the cohort with symptoms lasting more than 14 days (*p* = 0.038). In addition, patients in the acute cohort were significantly more likely to have critical limb ischemia (CLI, *p* < 0.001).

Study lesions included occluded bypass grafts in 262 patients (21.2%, 83 venous (6.7%) and 179 prosthetic (14.5%)), stent occlusions in 384 patients (31%), and 87 cases (7%) of an occluded Viabahn-endoprosthesis. Seventy-five patients (6.1%) had an occluded aneurysm.

A retrograde cross-over access was used in 882 cases (71.2%). In 80.6%, patients underwent rotational thrombectomy with the Rotarex^®^ thrombectomy system (Becton Dickinson, Franklin Lakes, NJ, USA). Stand-alone aspiration thrombectomy or in addition to rotational thrombectomy prior to CDT was performed in 350 cases (28.3%). In addition to rt-PA, 239 patients (19.3%) received abciximab and 129 (10.4%) alprostadil infusions. Details of these patients are shown in Tables S2 and S3 in the supplement. The details of the thrombolytic therapy are shown in Table 2.

### 3.2. Primary Endpoint

SAEs occurred in 511 (41.3%) of the patients (Table 3). Bleeding complications occurred in 314 patients (25.4%) with the most frequent bleeding at the access site (*n* = 153, 12.4%). In 107 cases (8.6%), there was a drop in hemoglobin following the procedure, but no active bleeding or hematoma was reported. Gastrointestinal bleeding occurred in 31 patients (2.5%) and bleeding at distant sites in 19 patients (1.5%). The remaining bleeding events were hematuria and pulmonary bleeding (*n* = 4, 0.3%).

There were 96 cases of reperfusion edema with or without compartment syndrome (7.8%), occurring more frequently in patients with acute ischemia (*n* = 56, 4.5%), as compared to patients with chronic PAD (*n* = 40, 3.2%, *p* = 0.121). A total of 82 patients with compartment syndrome (85.4%) had CLI and 84 patients (87.5%) needed a fasciotomy.

In total, 42 patients (3.4%) required amputations during the hospitalization (6 minor amputations, 22 below-the-knee amputations, and 14 above-the-knee amputations).

In total, 33 patients (2.7%) died during hospitalization; the causes of death are detailed in Table 4. The mean age of the deceased patients was higher (81.0 ± 9.0 years) than those of the overall cohort (69.0 ± 11.8 years, *p* < 0.001).

### 3.3. Secondary Endpoints

Thrombolysis was successful in 1177 cases (95.1%). Of the sixty-one patients who were not successfully lysed (4.9%), fifteen patients died (1.2%), twenty-two (1.8%) had a major amputation, nine (0.7%) were successfully treated in a further intervention and four patients (0.3%) underwent surgical therapy. Nine patients (0.7%) were treated conservatively. Two additional patients (0.2%) were transferred to another hospital due to a complication without information about the clinical outcome.

Multivariate logistic regression analysis identified age, abciximab and alprostadil usage, and lysis duration as predictors for SAEs. Preventives for the development of SAEs were a body mass index (BMI) between 25 and 30 or above 30 and a cumulative lytic agent dose between 12.5 and 25.0 mg (Table 5). Predictors for bleeding were age, alprostadil usage, and lysis duration. Preventives for the development of bleeding were a BMI above 30, diabetes, and a cumulative lytic agent dose between 12.5 and 25.0 mg (Table 6). Multivariate logistic regression analysis identified the use of abciximab as a predictor of reperfusion edema with or without compartment syndrome (Table 7).

Limb salvage rates in the acute ischemia group were 93.7%, 90.8%, and 88.1% after 6, 12, and 24 months, respectively. In the chronic ischemia group, it was 94.5%, 92.4%, and 89.6% after 6, 12, and 24 months, respectively (Table 8). One- and two-year TLR rates were 46% and 57.2% including those patients who underwent a second revascularization procedure after failed index procedure (Table 8). The mean time to TLR was 7.2 ± 6.2 months.

Compared to baseline, ABI and RBC were significantly improved at each time point during follow-up (Table 8).

## 4. Discussion

To date, this is the largest published study evaluating safety, as well as the technical and clinical outcomes of patients undergoing CDT with rt-PA for PAD indication. Primary treatment success was 95.1%, being significantly higher than that reported in previous studies. In a review study summarizing the outcome using different thrombolytic drugs, the pooled mean acute success rate is given as 74.9% [6]. The high technical success rate compared to other studies is most likely explained by lesion preparation with mechanical thrombectomy devices for re-establishing antegrade flow before CDT in the majority of cases. Rotational suction thrombectomy with the Rotarex^®^ thrombectomy catheter was performed in 80.6% of the cases, and syringe-based aspiration thrombectomy in 28.3%, partially combined with Rotarex^®^ thrombectomy. In previous studies using rt-PA for CDT, mechanical thrombectomy was not performed [10,11] or in a few cases only [12]. The additional use of a mechanical thrombectomy device and the associated reduction in thrombus burden may also explain the shorter lysis duration (17.6 ± 7.3 h) compared to former studies (approximately 25 h) [10,12,13].

However, despite the shorter lysis duration, the cumulative lysis dose was comparable to that in historic studies due to a higher hourly rt-PA dose administered in the present study [10,13].

Overall, peri- and post-procedural complication rate was as high as 41.3%, with bleeding being the most common complication of lysis therapy, in particular access site bleeding. A comparison to historic publications is difficult as the definition of bleeding complications are inconsistent or often not even stated. However, reported data on the incidence of bleeding complications were at least comparable to that reported in the present work [6,10,14]; access site bleeding complications range from 15.7% to 28.6% [10,14].

Following bleeding complications, compartment syndrome was the second most common complication. Reperfusion edema with or without compartment syndrome occurred in 7.7% of cases. Of these, 85.4% (in total 6.8%) had to undergo fasciotomy, being in the lower range of incidence reported in former studies (range, 7.3–9.9%) [10,12,15]. As in other studies, compartment syndrome occurred more frequently in patients with severe ischemia [10,15]. Critical ischemia leads to microcirculatory changes due to the activation of inflammatory mediators. Microvascular permeability increases, leading to an increased rate of transcapillary fluid leakage and further increasing intra-compartment pressure [16]. So far, the risk of developing compartment syndrome was mainly seen in acute ischemia with an incidence of only 1% in chronic critical limb ischemia [10,17]. In this study, both the incidence of reperfusion edema with or without compartment syndrome and the need for fasciotomy did not differ significantly between the acute and chronic symptom groups. The reason for the higher frequency of compartment syndrome in chronic ischemia may be the high proportion of vessel preparation procedures performed in this study, potentially resulting in device (mainly guidewire)-induced initially undetected local bleeding complications. These micro-bleedings may become significant during CDT, resulting in a critical increase in compartment pressure.

Thirty-three patients (2.7%) died during hospitalization in most cases related to the underlying disease or the peri-procedural complication. Even if this mortality rate is about half as high as reported in previous studies (5–5.4%) [12,14,18], CDT is not a benign therapy and should be used with caution.

For both SAEs and bleeding in particular, there were predictive and preventive factors identified. Increasing age is a predictor of both bleeding and SAE occurrence. To the best of our knowledge, there are no studies which determined age as a significant risk factor for complications following CDT. However, reservations regarding CDT in elderly patients exist [19]. In an American survey among vascular surgeons, 2186 members of the society of vascular surgery were contacted. A third of the surgeons do not apply an age restriction for performing a thrombolytic therapy. Other surgeons set an age limit, with nearly one-third (29%) rejecting lysis therapy in patients older than 80 years. The present analysis confirms these reservations. Only one of the patients who died within 30 days was under 75 years of age, and 63.6% were over 80 years of age.

Additional intra-arterial administration of other GP IIb/IIIa receptor antagonists and vasodilators increases the risk of SAEs, including bleeding complications. Accordingly, these drugs should only be used with increased caution.

Having a BMI of more than 30 was protective against bleeding. A possible explanation for the prevention of access site bleeding complications could be a more stable sheath position in the adipose tissue, potentially reducing motion-induced vessel wall trauma by the sheath. This assumption is supported by the finding that lysis duration, but not the cumulative dose of the lytic agent, was a predictor of SAEs and bleeding. The sheath positioned in the groin may cause increasing local vessel trauma over time.

The use of abciximab and prostaglandins was the only predictor for the incidence of reperfusion edema with or without compartment syndrome. The additional administration of vasodilators through continuous infusion, mainly used as consequence of peri-procedural low flow, may increase capillary leakage, promoting the development of the reperfusion edema, whereas abciximab as GP IIb/IIIa-RA may increase the risk of intramuscular bleeding complications.

Similar to previous studies, the acute combined minor and major amputation rate is high (3.4%), as well being high throughout the duration of the following disease course. Compared with other studies [6,12], the 1-year amputation rate in the present study is 8.4% lower than in historic studies, with a range of 13% to 14.8%. Previous lysis studies mainly included patients with acute ischemia, whereas in the present study, about half of the patients were included with chronic symptoms, which may explain the lower amputation rate in this study. However, in the present study, acute procedure-related complications and clinical outcomes during follow-up, such as amputation rate, ABI, RBC, as well as TLR rates, do not differ significantly between patients with acute and chronic symptoms.

Patients with unsuccessful CDT have an overall poor prognosis, mainly due to the (anatomical) complexity of their disease. Of the 61 patients in whom the index intervention, including CDT, was not successful, 13 patients were successfully treated either by surgery or endovascular treatment in a second staged intervention. Thirty-eight of those unsuccessful patients (62.3%), however, died or underwent amputation. The anatomical complexity of the disease of patients undergoing CDT is underscored by the high 1- and 2-year TLR-rate of 46% and 57.2%, including those patients with initially unsuccessful CDT. Kühn et al. report a 1-year reintervention rate of 27.4% after a primarily successful thrombolytic treatment [20]. As a consequence of the high restenosis and re-intervention rate, close follow-up and initiation of optimized secondary preventive measures should be recommended.

### Limitations

First, this is a retrospective study, even if the data were collected from a prospective database including consecutive patients. Secondly, no uniform lysis protocol was applied. Furthermore, the study does not include a control group consisting of alternative endovascular or surgical strategies excluding CDT.

## 5. Conclusions

CDT is an effective endovascular method for the treatment of thrombotic peripheral artery occlusions, regardless of the duration of the symptoms. However, it is associated with a significant acute and chronic morbidity and mortality rate, particularly in elderly patients. As such, close monitoring of patients undergoing CDT in an intensive care unit is indispensable and patients should be included in a close follow-up program. A prospective study should evaluate the most effective and safest regimen for CDT regarding lesion preparation, lytic agent dose, lysis duration, and co-drug administration.

## Figures and Tables

**Table 1:** Baseline characteristics.

	Total Cohort *n* = 1238	Cohort with Acute Symptoms *n* = 639	Cohort with Chronic Symptoms *n* = 599	*p*-Value
Sex				
Male	905 (73.1%)	457 (71.5%)	448 (74.8%)	0.109
Age				
Mean (years)	68.98 ± 11.82	69.56 ± 12.18	68.37 ± 11.42	0.038
Median (years)	69	70	69	
Range (years)	16–99	18–99	16–95	
Hypertension	983 (79.4%)	511 (80%)	472 (78.8)	0.330
Diabetes mellitus	423 (34.2%)	207 (32.4%)	216 (36.1%)	0.097
Hyperlipidemia	959 (77.5%)	485 (75.9%)	474 (79.1)	0.098
Tobacco use				
Current	501 (40.5%)	254 (39.7%)	247 (41.2%)	0.318
Former	328 (26.5%)	166 (26.0%)	162 (27.0%)	0.359
Overweight (BMI > 25)	765 (61.8%)	406 (63.5%)	359 (60.0%)	0.224
Coronary heart disease	460 (37.2%)	247 (38.7%)	213 (35.6%)	0.143
Cerebral vascular disease	132 (10.7%)	60 (9.4%)	72 (12.0%)	0.080
Chronic dialysis	30 (2.4%)	19 (3.0%)	11 (1.8%)	0.132
Rutherford–Becker class				
RBC 1	3 (0.2%)	1 (0.2%)	2 (0.3%)	0.476
RBC 2	25 (2.0%)	8 (1.3%)	17 (2.8%)	0.037
RBC 3	388 (31.3%)	139 (21.8%)	249 (41.6%)	<0.001
RBC 4	589 (47.6%)	414 (64.8%)	175 (29.2%)	<0.001
RBC 5	218 (17.6%)	70 (11.0%)	148 (24.7%)	<0.001
RBC 6	15 (1.2%)	8 (1.3%)	7 (1.2%)	0.551
Clinical categories of acute limb ischemia [9]				
I		441		
IIA		125		
IIB		62		
III		11		

**Table 2:** Procedural details of thrombolytic therapy.

	Total Cohort *n* = 1238	Cohort with Acute Symptoms *n* = 639	Cohort with Chronic Symptoms *n* = 599	*p*-Value
Abciximab administration	239 (19.3%)	107 (16.7%)	132 (22.0%)	0.011
rt-PA bolus	1238 (100%)			
Alprostadil infusion	129 (10.4%)	72 (11.3%)	57 (9.5%)	0.180
Ø time of thrombolytic infusion (h)	17.6 ± 7.3	17.6 ± 7.6	17.6 ± 7.0	0.498
Cumulative rt-PA dose (mg)	19.5 ± 9 Range: 2.0–92.5	19.4 ± 9.1 Range: 3.0–66.75	19.8 ± 9.0 Range: 2.0–92.50	0.210

**Table 3:** Complications until discharge.

	Total Cohort *n* = 1238	Cohort with Acute Symptoms *n* = 639	Cohort with Chronic Symptoms *n* = 599	*p*-Value
Bleeding	314 (25.4%)	156 (24.4%)	158 (26.4%)	0.233
Reperfusion edema/compartment syndrome	96 (7.8%)	56 (8.8%)	40 (6.7%)	0.121
Fasciotomy	84 (6.8%)	49 (7.7%)	35 (5.8%)	0.617
Amputation	42 (3.4%)	24 (3.8%)	18 (3.0%)	0.284
Others	26 (2.1%)	09 (1.4%)	17 (2.8%)	0.090
Death	33 (2.7%)	18 (2.8%)	15 (2.5%)	0.435

**Table 4:** Causes of death until discharge.

Patients	Death Cause
*n* = 11	Multi-organ failure secondary to critical ischemia.
*n* = 6	Cardiac
*n* = 4	Acute renal failure, rhabdomyolysis
*n* = 3	Bleeding/hemorrhagic shock
*n* = 2	Consequences of falls (cerebral hematoma and femur fracture)
*n* = 2	Carcinoma progression
*n* = 1	Intracranial bleeding
*n* = 1	Respiratory insufficiency due to pneumonia
*n* = 3	Transfer for further treatment, cause of death not known

**Table 5:** Univariable and multivariable logistic regression to identify risk factors for peri-procedural severe adverse events.

	Univariable Analysis			Multivariable Analysis		
	OR ^9^	95% CI ^10^	*p*-Value	Adj. ^11^ OR	95% CI	Adj. *p*-Value
**Age**						
Age	1.026	1.016; 1.037	0.000	1.025	1.014; 1.037	0.000
**Body-Mass-Index (BMI) ^1^**						
BMI 25–30	0.746	0.581; 0.958	0.022	0.723	0.556; 0.939	0.015
BMI > 30	0.331	0.233; 0.466	0.000	0.344	0.239; 0.491	0.000
**Comorbidities ^2^**						
Arterial hypertension	1.047	0.792; 1.389	0.748			
Hyperlipidemia	0.830	0.634; 1.087	0.174			
Diabetes mellitus	0.792	0.622; 1.007	0.058			
Bleeding history	1.239	0.804; 1.901	0.327			
Chronic dialysis	1.646	0.795; 3.449	0.179			
**Smoking status**						
Current smoker ^3^	0.686	0.527; 0.894	0.005	0.912	0.67; 1.244	0.561
Former smoker ^3^	0.647	0.48; 0.869	0.004	0.793	0.577; 1.089	0.153
**Vascular diseases ^4^**						
Cerebro-arterial disease	1.018	0.703; 1.465	0.923			
Former Insult	1.286	0.871; 1.896	0.203			
Coronary artery disease	0.960	0.759; 1.213	0.732			
PCI ^5^	0.896	0.695; 1.153	0.394			
ACVB ^6^	0.998	0.651; 1.516	0.993			
**Clinical Presentation and Intervention details**						
Chronic symptoms ^12,13^	1.010	0.805; 1.267	0.930			
Antegrade access	1.272	0.992; 1.628	0.057			
Rotational atherectomy	0.794	0.598; 1.055	0.111			
**Medication**						
Abciximab Usage	2.106	1.584; 2.806	0.000	2.107	1.534; 2.903	0.000
Alprostadil Usage	1.861	1.291; 2.694	0.001	1.553	1.04; 2.323	0.031
**Lysis characteristics**						
Lysis duration in h	1.025	1.01; 1.042	0.002	1.036	1.015; 1.057	0.001
rt-PA dose 12.5–25.0 mg ^7^	0.703	0.519; 0.953	0.023	0.628	0.447; 0.881	0.007
rt-PA dose > 25.0 mg ^8^	1.141	0.788; 1.652	0.486	0.656	0.403; 1.064	0.089

^1^ Reference BMI: < 25, ^2^ Reference: Absence of specific comorbidity, ^3^ Reference: Non-smoker, ^4^ Reference: Absence of mentioned vascular diseases, ^5^ Percutaneous coronary intervention, ^6^ Aorto-coronary venous bypass, ^7,8^ Reference: Dosage < 12.5 mg rt-PA, ^9^ Odds ratio, ^10^ 95% confidence interval, ^11^ adjusted, ^12^ Chronic symptoms (Symptom onset > 14 days), ^13^ Reference: Acute symptoms (Symptom onset < 14 days).

**Table 6:** Univariable and multivariable logistic regression to identify risk factors for peri-procedural major bleeding events.

	Univariable Analysis			Multivariable Analysis		
	OR ^9^	95% CI ^10^	*p*-Value	Adj. ^11^ OR	95% CI	Adj. *p*-Value
**Age**						
Age	1.023	1.013; 1.034	0.000	1.025	1.013; 1.037	0.000
**Body-Mass-Index (BMI) ^1^**						
BMI 25–30	0.732	0.567; 0.943	0.016	0.784	0.598; 1.028	0.078
BMI > 30	0.291	0.198; 0.42	0.000	0.335	0.225; 0.492	0.000
**Comorbidities ^2^**						
Arterial hypertension	0.996	0.749; 1.332	0.981			
Hyperlipidemia	0.912	0.692; 1.204	0.512			
Diabetes mellitus	0.673	0.523; 0.865	0.002	0.737	0.56; 0.968	0.029
Bleeding history	1.031	0.655; 1.597	0.894			
Chronic dialysis	0.651	0.27; 1.418	0.303			
**Smoking status**						
Current smoker ^3^	0.747	0.57; 0.978	0.034	0.979	0.714; 1.346	0.897
Former smoker ^3^	0.602	0.441; 0.817	0.001	0.751	0.538; 1.047	0.092
**Vascular diseases ^4^**						
Cerebro-arterial disease	1.115	0.764; 1.613	0.567			
Former Insult	1.269	0.851; 1.878	0.237			
Coronary artery disease	0.761	0.596; 0.971	0.028	1.062	0.679; 1.646	0.789
PCI ^5^	0.712	0.545; 0.928	0.012	0.727	0.455; 1.171	0.186
ACVB ^6^	0.926	0.592; 1.424	0.732			
**Clinical Presentation and Intervention details**						
Chronic symptoms ^12,13^	0.995	0.788; 1.255	0.964			
Antegrade access	1.134	0.878; 1.46	0.334			
Rotational atherectomy	1.011	0.755; 1.361	0.941			
**Medication**						
Abciximab Usage	1.481	1.109; 1.973	0.007	1.371	0.991; 1.893	0.055
Alprostadil Usage	1.912	1.324; 2.761	0.001	1.749	1.171; 2.613	0.006
**Lysis characteristics**						
Lysis duration in h	1.027	1.011; 1.044	0.001	1.033	1.012; 1.054	0.002
rt-PA dose 12.5–25.0 mg ^7^	0.647	0.475; 0.882	0.006	0.590	0.418; 0.834	0.003
rt-PA dose > 25.0 mg ^8^	1.120	0.772; 1.628	0.551	0.739	0.451; 1.206	0.227

^1^ Reference BMI: < 25, ^2^ Reference: Absence of specific comorbidity, ^3^ Reference: Non-smoker, ^4^ Reference: Absence of mentioned vascular diseases, ^5^ Percutaneous coronary intervention, ^6^ Aorto-coronary venous bypass, ^7,8^ Reference: Dosage < 12.5 mg rt-PA, ^9^ Odds ratio, ^10^ 95% confidence interval, ^11^ adjusted, ^12^ Chronic symptoms (Symptom onset > 14 days), ^13^ Reference: Acute symptoms (Symptom onset < 14 days).

**Table 7:** Univariable and multivariable logistic regression to identify risk factors for post-procedural reperfusion edema/compartment syndrome.

	Univariable Analysis			Multivariable Analysis		
	OR ^9^	95% CI ^10^	*p*-Value	Adj. ^11^ OR	95% CI	Adj. *p*-Value
**Age**						
Age	1.026	1.016; 1.037	0.000	1.008	0.988; 1.029	0.431
**Body-Mass-Index (BMI) ^1^**						
BMI 25–30	0.746	0.581; 0.958	0.022	0.858	0.535; 1.376	0.524
BMI > 30	0.331	0.233; 0.466	0.000	0.933	0.506; 1.661	0.818
**Comorbidities ^2^**						
Arterial hypertension	1.047	0.792; 1.389	0.748			
Hyperlipidemia	0.830	0.634; 1.087	0.174			
Diabetes mellitus	0.792	0.622; 1.007	0.058			
Bleeding history	1.239	0.804; 1.901	0.327			
Chronic dialysis	1.646	0.795; 3.449	0.179			
**Smoking status**						
Current smoker ^3^	0.686	0.527; 0.894	0.005	1.185	0.686; 2.071	0.546
Former smoker ^3^	0.647	0.48; 0.869	0.004	1.089	0.615; 1.915	0.767
**Vascular diseases ^4^**						
Cerebro-arterial disease	1.018	0.703; 1.465	0.923			
Former Insult	1.286	0.871; 1.896	1.286			
Coronary artery disease	0.960	0.759; 1.213	0.732			
PCI ^5^	0.896	0.695; 1.153	0.394			
ACVB ^6^	0.998	0.651; 1.516	0.993			
**Intervention details**						
Chronic symptoms ^12,13^	1.010	0.805; 1.267	0.930			
Antegrade access	1.272	0.992; 1.628	0.057			
Rotational atherectomy	0.794	0.598; 1.055	0.111			
**Medication**						
Abciximab Usage	2.106	1.584; 2.806	0.000	1.718	1.03; 2.805	0.034
Prostavasin Usage	1.861	1.291; 2.694	0.001	1.346	0.703; 2.432	0.345
**Lysis characteristics**						
Lysis duration in h	1.025	1.01; 1.042	0.002	1.014	0.983; 1.045	0.356
rt-PA dose 12.5–25.0 mg ^7^	0.703	0.519; 0.953	0.023	0.924	0.509; 1.77	0.803
Rt-PA dose > 25.0 mg ^8^	1.141	0.788; 1.652	0.486	1.310	0.589; 2.966	0.510

^1^ Reference BMI: < 25, ^2^ Reference: Absence of specific comorbidity, ^3^ Reference: Non-smoker, ^4^ Reference: Absence of mentioned vascular diseases, ^5^ Percutaneous coronary intervention, ^6^ Aorto-coronary venous bypass, ^7,8^ Reference: Dosage 12.5 mg rt-PA, ^9^ Odds ratio, ^10^ 95% confidence interval, ^11^ adjusted, ^12^ Chronic symptoms (Symptom onset > 14 days), ^13^ Reference: Acute symptoms (Symptom onset < 14 days).

**Table 8:** Clinical outcome during follow-up.

	Total Cohort *n* = 1238	Cohort with Acute Symptoms *n* = 639	Cohort with Chronic Symptoms *n* = 599	*p*-Value
**ABI**				
Baseline (*n* = 627)	0.32 ± 0.33	0.25 ± 0.33	0.37 ± 0.33	<0.001
Post-procedure (*n* = 935)	0.91 ± 0.31 (<0.001)	0.93 ± 0.30	0.90 ± 0.31	0.094
6 months (*n* = 555)	0.79 ± 0.36 (<0.001)	0.77 ± 0.34	0.81 ± 0.39	0.076
12 months (*n* = 495)	0.77 ± 0.40 (<0.001)	0.75 ± 0.41	0.80 ± 0.39	0.117
24 months (*n* = 423)	0.76 ± 0.39 (<0.001)	0.77 ± 0.43	0.75 ± 0.35	0.382
**RBC**				
Baseline (*n* = 1238)	3.8 ± 0.8	3.9 ± 0.65	3.8 ± 0.89	0.014
6 months (*n* = 647)	2.6 ± 1.6 (<0.001)	2.5 ± 1.6	2.7 ± 1.7	0.147
12 months (*n* = 581)	2.7 ± 1.6 (<0.001)	2.6 ± 1.6	2.8 ± 1.7	0.140
24 months (*n* = 507)	2.5 ± 1.6 (<0.001)	2.5 ± 1.5	2.5 ± 1.6	0.433
**TLR rate**				
6 months (*n* = 1005)	329 (32.7%)	178 (33.5%)	151 (31.9%)	0.303
12 months (*n* = 1005)	462 (46%)	250 (47%)	212 (44.7%)	0.296
24 months (*n* = 1005)	575 (57.2%)	307 (57.8%)	268 (56.5%)	0.365
**Amputation**				
6 months (*n* = 937)	59 (6.3%)	32 (6.5%)	27 (6.1%)	0.465
12 months (*n* = 844)	74 (8.8%)	42 (9.4%)	32 (8.1%)	0.287
24 months (*n* = 762)	87 (11.4%)	49 (12.1%)	38 (10.6%)	0.295
Major amputation	81 (6.5%)	47 (7.4)	34 (5.7%)	0.140
Minor amputation	6 (0.5%)	2 (0.3%)	4 (0.7%)	0.314

## Data Availability

The data presented in this study are available on request from the corresponding author.

## Supplementary Materials

The following supporting information can be downloaded at: https://www.mdpi.com/article/10.3390/jcm13195732/s1, Table S1. Definition adverse and serious adverse event [7]; Table S2. Differences between patients treated with and without abciximab; Table S3. Differences between patients treated with and without alprostadil infusion.

## Abbreviations

ABI	ankle–brachial index
BMI	Body mass index
CDT	catheter-directed thrombolysis
PAD	peripheral artery disease
SAE	serious adverse event
RBC	Rutherford–Becker class
TLR	target lesion revascularization
