# Supplementary material for: Catheter-Directed Thrombolysis in the Management of Thrombotic Peripheral Artery Occlusions—Acute and Mid-Term Clinical Outcomes
Source: J Clin Med. 2024 Sep 26;13(19):5732. doi: 10.3390/jcm13195732 (PMC11477182; doi:10.3390/jcm13195732)
Supplement: Supplementary file 1 [file jcm-13-05732-s001.zip › jcm-3181860-supplementary.pdf]

Supplement

Table S1. Definiton adverse and serious adverse event [7]

**ISO 14155:2020**	
**Adverse event (AE) (3.2)**<br>Untoward medical occurrence, unintended disease or injury, or untoward clinical signs (including abnormal laboratory findings) in subjects, users or other persons, whether related or not related to the investigational device and whether anticipated or unanticipated<br><br>Note 1 to entry: This definition includes events related to the investigational medical device or the comparator<br><br>Note 2 to entry: This definition includes events related to the procedures involved.<br><br>Note 3 to entry: for users or other persons, this definition is restricted to events related to the use of investigational medical devices or comparators.	**Serious adverse event (SAE) (3.45)**<br>Adverse event that led to any of the following<br><br>a) death,<br><br>b) serious deterioration in the health of the subject, users, or other persons as defined by one or more of the following:<br>1. a life-threatening illness or injury, or<br>2. a permanent impairment of a body structure or a body function including chronic diseases, or<br>3. in-patient or prolonged hospitalization, or<br>4. medical or surgical intervention to prevent life-threatening illness or injury, or permanent impairment to a body structure or a body function,<br><br>c) fetal distress, foetal death, a congenital abnormality, or birth defect including physical or mental impairment<br><br>Note 1 to entry: Planned hospitalization for a preexisting condition, or a procedure required by the CIP, without serious deterioration in health, is not considered a serious adverse event.

Table S2. Differences between patients treated with and without abciximab

	Cohort with abciximab n = 239	Cohort without abciximab n = 999	p-value
Chronic symptoms	132 (55.2)	467 (46.7)	0.011
Coronary heart disease	102 (42.7)	358 (35.8)	0.030
RBC 4	94 (39.3)	495 (49.5)	0.003
RBC 5	59 (24.7)	159 (15.9)	0.001
Alprostadil infusion	53 (22.2)	76 (7.6)	<0.001

Table S3. Differences between patients treated with and without alprostadil infusion

	Cohort with Alprostadil infusion n = 129	Cohort without Alprostadil infusion n = 1109	p-value
Mean Age (years)	65.6±14.9	69.4±11.4	0.001
Hypertension	93 (72.1)	890 (80.3)	0.022
RBC 3	28 (21.7)	360 (32.5)	0.007
RBC 5	46 (35.7)	172 (15.5)	<0.001
Abciximab administration	53 (41.1)	180 (16.8)	<0.001
